# Treatment outcomes and prognostic factors for non- malignancy associated secondary hemophagocytic lymphohistiocytosis in children

**DOI:** 10.1186/s12887-020-02178-7

**Published:** 2020-06-09

**Authors:** Hua Pan, Gaoyan Wang, Enben Guan, Liang Song, Aiqin Song, Xiaodan Liu, Zhi Yi, Li-rong Sun

**Affiliations:** Department of Paediatric Medical Center, Affiliated Hospital of Qingdao University, 16 Jiangsu Road, Qingdao, 266003 Shandong China

**Keywords:** Hemophagocytic lymphohistiocytosis, Prognostic factor, risk stratification

## Abstract

**Background:**

**S**econdary hemophagocytic lymphohistiocytosis (HLH) is a rare hyperinflammatory syndrome that requires prompt diagnosis and appropriate treatment. A risk-stratification model that could be used to identify high-risk pediatric patients with HLH who should be considered for second-line therapies, including salvage regimens and allogeneic hematopoietic cell transplantation (HCT), was developed.

**Methods:**

The medical records of 88 pediatric patients (median age 1.4 years, range 0.2–15 years) with non-malignancy associated secondary HLH were retrospectively reviewed. Treatment strategies included dexamethasone, etoposide, and cyclosporine.

**Results:**

Survival analysis showed HLH patients with infections other than Epstein-Barr virus (EBV) and unknown causes experienced better 5-year overall survival (OS) than patients with HLH due to autoimmune disease, EBV or immunodeficiency (76% vs. 65, 33.3, 11%, *p* < 0.001). On multivariate analysis, among all patients, non-response at 8 weeks was the most powerful predictor of poor OS. When treatment response was excluded, hemoglobin < 60 g/L and albumin < 25 g/L at diagnosis were associated with poor OS. In patients with EBV-HLH, hemoglobin < 60 g/L at diagnosis was associated with poor OS. A prognostic risk score was established and weighted based on hazard ratios calculated for three parameters measured at diagnosis: hemoglobin < 60 g/L (2 points), platelets < 30 × 10^9^/L (1 point), albumin < 25 g/L (2 points). Five-year OS of low-risk (score 0–1), intermediate-risk (score 2), and poor-risk (score ≥ 3) patients were 88, 38, and 22%, respectively (*p* < 0.001).

**Conclusions:**

These findings indicate that clinicians should be aware of predictive factors at diagnosis and consider 8-week treatment response to identify patients with high-risk of disease progression and the need for second-line therapy and allogeneic HCT.

## Background

Hemophagocytic lymphohistiocytosis (HLH) is a potentially fatal condition of genetic or functional hypercytokinemia that is characterized by uncontrolled proliferation of activated lymphocytes and macrophages. HLH is associated with a wide spectrum of signs and symptoms, including fever, hepatosplenomegaly, pancytopenias, hypertriglyceridemia, hypofibrinogenemia, neurological symptoms and pathological findings of hemophagocytosis in the bone marrow or other organs [1]. HLH is a life-threatening condition. Prompt diagnosis and initiation of treatment with dexamethasone and etoposide are essential [1]. Treatment of HLH can be associated with high morbidity and mortality, including severe sepsis and multi-organ failure in relapsed/refractory patients [1].

There are two major subtypes of HLH, which have been classified as “primary HLH” and “secondary HLH” based on family history, and the identification of a series of genetic variations that are responsible for the decreased functions of T/NK cells, such as PRF1/STXBP2/ITK [2]. Recently, Jordan et al. proposed two concepts to clarify how HLH is diagnosed and treated: within the broader syndrome of HLH, “HLH disease” should be distinguished from “HLH disease mimics” and HLH subtypes should be categorized by specific etiologic associations, not an ambiguous dichotomy of “primary” and “secondary” [3].

Traditionally, secondary HLH is considered a rare hyperinflammatory syndrome that occurs in individuals without a family history or underlying genetic defect. Secondary HLH may be triggered by infections, rheumatologic diseases, malignancy, acquired immune deficiency states, and drugs [4]. Diagnosis and initiation of treatment for patients with secondary HLH is difficult because signs and symptoms overlap with several chronic conditions, including sepsis, multi-organ dysfunction, and progression of malignancies or rheumatic disease [5, 6].

There remains an umet need to increase clinicians’ knowledge of HLH. In particular clinicians must be aware of factors that are predictive of treatment response and survival in patients with HLH. Previous studies showed that indicators such as age, associated infection, cerebrospinal fluid pleocytosis, and family history were not associated with treatment outcomes [7], while hyperbilirubinaemia, hyperferritinaemia, and cerebrospinal fluid pleocytosis (> 100 × 10^6^/L) at diagnosis and thrombocytopenia and hyperferritinaemia 2 weeks after the initiation of therapy were significantly associated with adverse outcomes [8].

The objectives of this retrospective study were to evaluate treatment response and mortality and identify predictive factors for treatment response and survival in children with non-malignancy associated secondary HLH. A risk-stratification model that could be used to identify high-risk pediatric patients with HLH who should be considered for second-line therapies, including salvage regimens and allogeneic HCT, was developed.

## Methods

### Study subjects

Children diagnosed with HLH between 2005 and 2018 according to HLH-2004 criteria (fever, splenomegaly, bicytopenia, hypertriglyceridemia and/or hypofibrinogenemia, hemophagocytosis, ferritin ≥500 μg/L, low/absent NK-cell activity, and soluble CD25 [sIL-2r] [≥2400 U/ml], an affected sibling, and/or a molecular diagnosis based on familial hemophagocytic syndrome (FHL)-causative genes were eligible for his study [4]. The majority of patients satisfied at least 5/6 of these criteria as NK-cell activity and soluble CD25 were not evaluated. Exclusion criteria were 1) diagnostic criteria not met or 2) malignancy-associated HLH (MA-HLH). This research was conducted in accordance with the guidelines of the Ethics Committee of the Affiliated Hospital of Qingdao University. Consent was obtained from a parent or guardian on behalf of any participants under the age of 16 years.

### Parameters associated with HLH

The medical records of the included patients were retrospectively reviewed. Pretreatment complete blood cell (CBC) count, levels of fibrinogen and albumin, and prothrombin time (PT) were extracted from serial laboratory analyses.

Clinical outcomes were assessed based on the highest ferritin, aspartate aminotransferase (AST), alanine aminotransferase (ALT), total bilirubin, lactate dehydrogenase (LDH), and triglyceride levels recorded during the 4 weeks after treatment initiation.

Epstein-Barr virus (EBV)-association was evaluated by viremia (including low level chronic viremia) and symptomatology of EBV infection, serologic tests (EBV early antigen [EA], EBV nuclear antigen [EBNA], EBV viral capsid antigen [VCA] IgG/IgM) and measuring EBV DNA level using real time polymerase chain reaction (RT-PCR).

### Treatments

Treatment strategies were based on the HLH-94/04 protocol, and included dexamethasone, etoposide, and cyclosporine [9, 10]. If complete response was achieved at week 8, patients were provided maintenance therapy, while patients with known familial disease or persistent nonfamilial disease proceeded to continuation therapy.

Treatment response at 4 weeks and 8 weeks after treatment, and dynamic changes during the 8 weeks of treatment were evaluated. Complete response was defined as resolution of all clinical signs and symptoms, CBC recovery, and normalization of abnormal laboratory findings associated with HLH. Partial response was defined as either CBC recovery or normalization of laboratory findings. Progressive disease was defined as persistence of cytopenia and abnormal laboratory findings.

Early stable responders were defined as patients with a complete response at both 4 and 8 weeks after treatment. Late stable responders were defined as patients who failed to achieve complete response at 4 weeks after treatment but showed a continuous response until 8 weeks after treatment. Unstable responders were defined as patients who showed a transient response and eventually progressed to 8 weeks after treatment. Non-responders were defined as patients exhibiting no response (See Supplemental methods for Statistical analysis).

### Statistical methods

The clinical characteristics and laboratory test results of children were described statistically. The normal distribution of measurement data is represented as mean ± standard deviation; the non normal distribution is represented by median (minimum maximum); the count data is represented by quantity and percentage. The total survival time was estimated using Kaplan Meier method, defined as the time from HLH diagnosis to death due to any cause. The total survival time of each group was compared by log rank test. Single factor and multivariate Cox analysis were used to evaluate the prognostic factors of HLH. In all the analyses, *P* < 0.05 was considered as statistically significant. SPSS software 22 was used for all statistical analysis.

## Results

### Baseline characteristics

The medical records of 88 patients (median age 1.4 years, range 0.2–15 years; mean age 3.5 years) with non-malignancy associated secondary HLH were retrospectively reviewed. All patients were negative on mutation analysis of all FHL-related genes. Causes of HLH were: EBV-associated (*n* = 24: recent primary infection *n* = 21, chronic active EBV infection *n* = 3), unknown cause (*n* = 25), infection other than EBV (*n* = 20:cytomegalovirus *n* = 10, brucella *n* = 3, *Staphylococcus aureus n* = 2, *Streptococcus pneumoniae n* = 2, Mycoplasm *n* = 1, parvovirus *n* = 1, herpes simplex *n* = 1), autoimmune disease (*n* = 13: systemic lupus erythematosus *n* = 4, juvenile rheumatoid arthritis *n* = 5, dermatomyositis *n* = 1, Kawasaki disease *n* = 2, anaphylactoid purpura *n* = 1), or immunodeficiency (*n* = 6: chronic granuloma *n* = 4, humoral immunodeficiency *n* = 1, combined immunodeficiency *n* = 1). Baseline characteristics and treatment response for the included patients are summarized in Table 1. Fever was observed in all patients and 49 (55.7%) patients had splenomegaly. Peak ferritin levels were reached at a median of 7 days after initiation.

**Table 1:** Baseline characteristics and treatment outcomes of included patients (*n* = 88)

Characteristic	Value
Gender (male), %	50 (56.8%)
Age, years old	1.4 (0.2–15)
Fever	88 (100%)
CMV %	13 (14.8%)
Leukocyte	3.22 (0.25–36.47)
Neutrophil	1.195 (0.05–11.86)
Platelet	67.00 (7–562)
Hemoglobin	86.5 (31–131)
Fibrinogen	1.29 (0.27–4.56)
Albumin	29.27 ± 5.61
Ferritin	3564.5 (132.0–591,123.0)
AST	249.15 (21.0–9420.0)
ALT	207.50 (8.0–4953.0)
LDH	944.5 (156.0–5311.0)
TG	3.34 (0.39–13.56)
CRP	15.05 (0.25–161.54)
GGT	207.5 (7.0–1550.0)
D-dimer	2489.0 (129.0–35,600.0)
DIC %	28 (31.8%)
Splenomegaly %	49 (55.7%)
Jaundice %	34 (38.6%)
Bone marrow involvement %	51 (58.0%)
Hepatomegaly %	65 (73.9%)
Ascites %	21 (23.9%)
Lymphadenopathy % CR achievement after treatments	28 (31.8%) 58 (65.9%)
Therapy	
HLH94/2004based therapy	34 (38.6%)
Steroid + IVIG	8 (9.1%)
Steroid	22 (25%)
Only observation	23 (26.1%)
Hematopoietic stem cell transplantation	1 (1.1%)
Causes of HLH	56 (76.7%)
EBV-associated	30 (33.7%)
Immunodeficiency	6 (6.7%)
Autoimmune disease (AID)	13 (14.6%)
Unknown cause and infection other than EBV	40 (44.9%)
Treatment response at 8 weeks	
Early stable responder	29 (33.3%)
Late stable responder	28 (32.2%)
Unstable responder	17 (19.5%)
Non-responder	13 (14.9%)

Abbreviations: *HLH* hemophagocytic lymphohistiocytosis, *AST* aspartate aminotransferase, *ALT* alanine transferase, *LDH* lactate dehydrogenase, *CRP* C-reactive protein, *DIC* disseminated intravascular coagulation, *EBV* Epstein-Barr virus, *CR* complete remission, *EBV* Epstein-Barr virus, *TG* triglyceride, *GGT* gamma-glutamyl transpeptidase

### Treatment outcomes

**Fig. 1:**
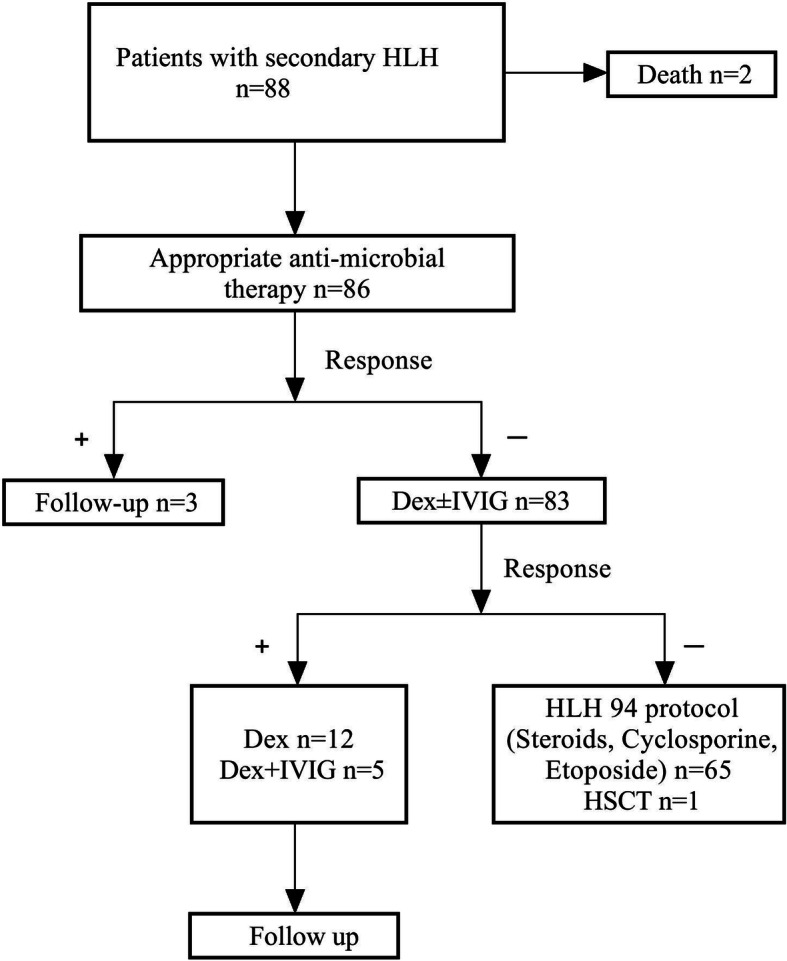
Flow-chart showing the treatment pathway

A total of 83/88 (94.3%) patients were treated. 65/88 (73.8%) (unknown cause and infection other than EBV *n* = 45, EBV infection *n* = 24, autoimmune disease *n* = 13, immunodeficiency *n* = 6) patients were administered therapy according to the HLH94 protocol. 5/88 (5.6%) patients were not treated. Of these, 3 patients had no signs of HLH activity and 2 patients died. 12/88 (13.6%) patients were administered dexamethasone alone. 5/88 (5.6%) patients were administered dexamethasone plus gamma globulin. 1/88 (1.1%) patient underwent HCT (Fig. 1). The frequency and dose of etoposide was not reduced for patients with substantially altered organ function or who were in a generally poor condition.

57/88 (64.7%) patients achieved a complete response; of these, 30 patients achieved complete response at 4 weeks after treatment and 27 achieved complete response at 8 weeks or later. Three patients relapsed; of these, 1 patient relapsed 8 weeks after treatment, 1 patient relapsed 4 months after treatment, and 1 patient relapsed 12 months after treatment. Relapsed patients had EBV-HLH, and were treated with rituximab (*n* = 1), alemtuzumab (*n* = 1), or allogeneic-HCT (*n* = 1) according to the HLH-94 protocol. Regarding dynamic response, 32/88 (36.4%) patients were early stable responders, 28/88 (31.8%) were late stable responders, 14/88 (15.9%) were unstable responders, and 13/88 (14.7%) were primary refractory non-responders.

### Survival outcomes according to treatment response

**Fig. 2:**
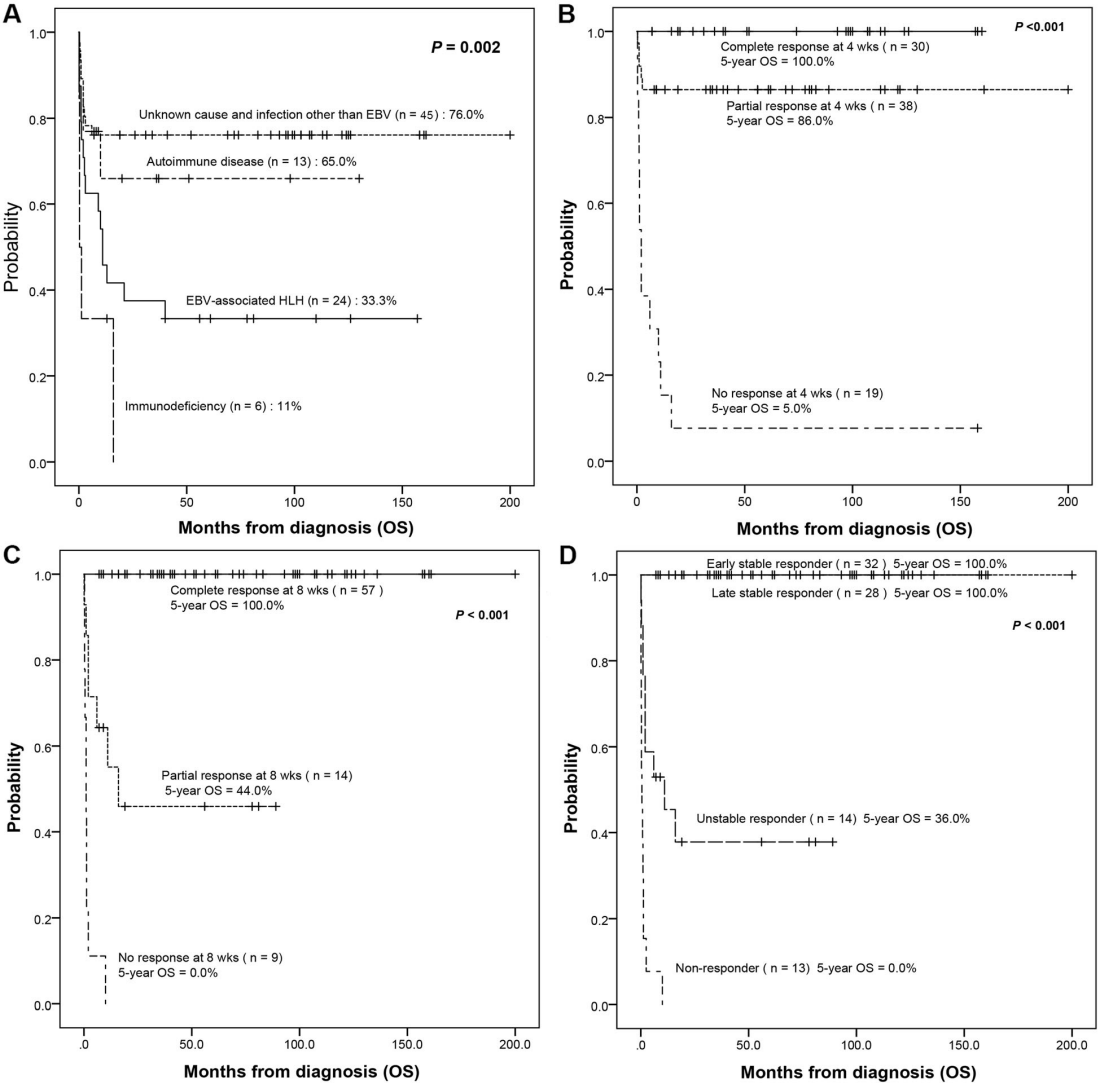
Survival outcomes according to the cause of secondary HLH and treatment response. a The cause of secondary HLH. **b** Treatment response at 4 weeks. **c** Treatment response at 8 weeks. **d** Dynamic treatment responses at 8 weeks

The overall mortality for all patients was 35.2% (31/88). 5-year OS rates for patients with an unknown cause and infections other than EBV due to autoimmune disease were 76.0 and 65%, respectively, and higher than in patients with HLH from other causes (Fig. 2a). Among all patients, 5-year OS rates for patients who achieved complete response at 4 weeks (*n* = 30) and 8 weeks (*n* = 57) were each 100%. 5-year OS rates for patients who achieved partial response at 4 weeks (*n* = 38) and 8 weeks (*n* = 14) were 86 and 44.0%, respectively (Fig. 2b-c). 5-year OS rates for early stable responders and late stable responders were each 100% (Fig. 2d).

### Prognostic factors and risk stratification

On univariate survival analysis, hemoglobin level, PLT, and albumin level at diagnosis, and dynamic treatment response were significantly correlated with survival outcomes. On multivariate analysis, non- response at 8 weeks was the most powerful predictor of poor OS. When treatment response was excluded, hemoglobin< 60 g/L and albumin< 25 g/L at diagnosis were associated with poor OS (Table 2). In patients with EBV-HLH, hemoglobin < 60 g/L at diagnosis was associated with poor OS.

**Table 2:** Univariate and multivariate analysis of parameters affecting OS in non-malignancy associated HLH

	Univariate analysis		Multivariate analysis			
			Treatment response included		Treatment response excluded	
	5-year OS	*P*	HR(95%CI)	*P*	HR(95%CI)	*P*
Age < 1 years old	80.0% VS (69.0%)	0.425				
EBV-associated	79.0% VS (67.0%)	0.262				
Hemoglobin < 60 g/L	25.0% VS (77.0%)	**< 0.001**			3.22 (2.01–19.28)	**0.002**
Platelet < 30 × 10^9/L	36.0% VS (79.0%)	**< 0.001**			2.15 (0.88–5.24)	0.093
ALT > 200 U/L	73.0% VS (71.0%)	0.935				
TG > 2.5 mg/dl	76.0% VS (62.0%)	0.173				
Fibrinogen < 1.5	66.0% VS (80.0%)	0.170				
Ferritin > 10,000	75.0% VS (72.0%)	0.744				
Albumin < 25 g/L	33.0% VS (82.0%)	**< 0.001**			7.71 (3.10–19.19)	**< 0.001**
Dynamic treatment response		**0.010**		**0.010**		
Early stable responder	100%		0.00			
Late stable responder	100%		0.00			
Unstable responder	36%		0.21 (0.08–0.52)			
Non-responder	0%		1.0			

Abbreviations: *OS* overall survival, *HLH* hemophagocytic lymphohistiocytosis, *HR* hazard ratio, *EBV* Epstein-Barr virus, *ALT* alanine transferase, *TG* triglyceride

**Fig. 3:**
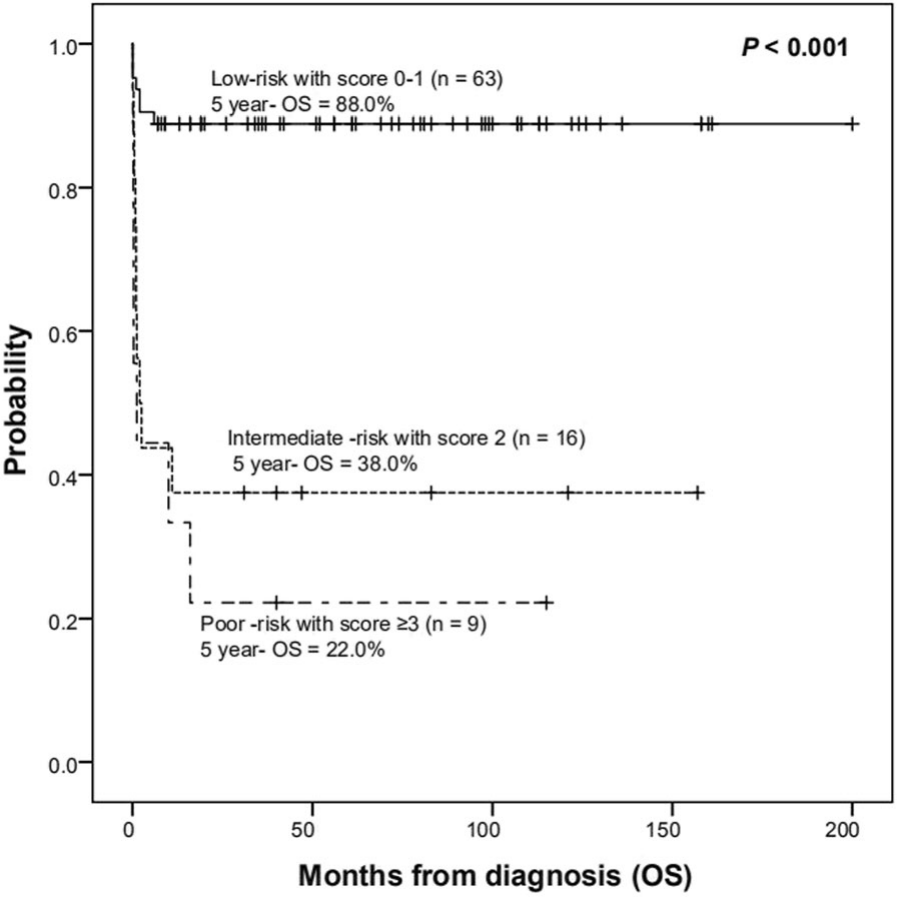
Survival outcomes according to HLH risk-score (low-risk [score 0–1], intermediate-risk [score 2], and high-risk [score ≥ 3])

A prognostic risk score was established and weighted based on hazard ratios calculated for three parameters measured at diagnosis: hemoglobin < 60 g/L (2 points), platelets < 30 × 10^9^/L (1 point), albumin < 25 g/L (2 points). Five-year OS of low-risk (score 0–1), intermediate-risk (score 2), and poor-risk (score ≥ 3) patients were 88, 38, and 22%, respectively (Fig. 3).

## Discussion

This study analyzed clinical symptoms, treatment response and prognostic factors in pediatric patients with secondary HLH after excluding MA-HLH. Consistent with a previous report [11], hepatomegaly (73.9%) was more prevalent than splenomegaly (55.7%) in our patient population, although hepatomegaly was not part of the diagnostic criteria included in the HLH-04 clinical trial.

EBV infection is a common cause of HLH. Evidence suggests that the presence of VCA IgG and VCA IgM in the absence of EBNA IgG is indicative of an acute EBV infection and the presence of VCA IgG and EBNA IgG in the absence of VCA IgM shows a previous infection [12, 13]. In the present study, evaluation of EBV-specific antibodies (EBV VCA IgM, IgG and EBNA-1 IgG) and serous EB DNA virus load (> 10^5^/copies) was used to confirm an EBV diagnosis. In our pediatric population, patients with EBV-HLH showed a worse treatment response and progressive OS (33.3%) than patients with secondary HLH due to other causes, except immunodeficiency, and patients with HLH caused by EBV infection had a very poor prognosis. Consistent with this, a study in adult patients with HLH reported that EBV was associated with poor survival outcomes, and the overall response to treatment at 4 weeks was similar in patients with HLH caused by EBV, autoimmune disease, other infections, or unknown causes, but decreased in the EBV group at 8 weeks [14]. Similarly, survival in pediatric patients with EBV-induced secondary HLH/macrophage activation syndrome (MAS) associated with rheumatic and nonrheumatic conditions was 50% [15].

Etoposide is very important for the treatment of HLH in patients who do not achieve remission with dexamethasone and gamma globulin. Etoposide, a chemotherapeutic agent, has high specificity against T-cell proliferation and cytokine secretion in mice [16]. Abnormal liver function in children with hemophagocytic syndrome is a cytokine storm syndrome. Therefore, the frequency and dose of etoposide was not reduced in patients with substantially altered organ function or who were in a generally poor condition, and liver function returned to normal with chemotherapy and remission.

Urgent preparation for allogeneic HCT should be considered for high-risk patients at HLH diagnosis [17]. In the present study, prognosis was very poor in patients with HLH caused by autoimmune diseases and immunodeficiency. HLH that results from rheumatic or other systemic diseases, including Still’s disease and sarcoidosis, is termed MAS [18–20]. In our cohort, 13 patients had HLH caused by autoimmune disease, including systemic lupus erythematosus, juvenile rheumatoid arthritis, dermatomyositis, Kawasaki disease, and anaphylactoid purpura, and the survival rate was 65%. In a previous study, 100% (*n* = 13) of pediatric patients with secondary HLH/MAS and underlying systemic juvenile idiopathic arthritis survived after treatment with a recombinant human interleukin-1 receptor antagonist [15].

There were 6 patients with HLH caused by immunodeficiency, including chronic granuloma, X-linked lymphocyte proliferative diseases (XLP), and humoral immune deficiency. In addition to abnormalities in cytotoxic granules and lysosomes, various primary immune deficiency disorders (PID) other than familial hemophagocytic lymphohistiocytosis (FHL) or XLP disorders have been identified among patients suffering from HLH [21]. In one report, patients with primary immunodeficiencies other than cytotoxicity defects or XLP disorders presenting with conditions fulfilling current criteria for HLH had chronic granulomatous disease with hemophagocytic episodes mainly associated with bacterial infections. The authors proposed that HLH syndrome in chronic granulomatous disease not only reflects an impaired response to infection, but also a genetic predisposition to the inflammatory reaction [22].

In the present study, 5-year OS rates for early stable responders and late stable responders were each 100%, indicating that patients can experience long-term survival if they fail to achieve a complete response at 4 weeks after treatment but exhibit a continuous and stable response until 8 weeks. The 5-year OS rate for patients who achieved partial response at 8 weeks was low at 44.0%. On multivariate analysis, non- response at 8 weeks was the most powerful predictor of poor OS. When treatment response was excluded, hemoglobin < 60 g/L and albumin < 25 g/L at diagnosis were associated with poor OS. In previous reports in adult patients with HLH, one study associated hypofibrinogenemia (≤150 mg/dl), fibrinogen ≤200 mg/dl (*P* = 0.04), and prothrombin time > 50% with increased mortality [23], while another showed that patients with disseminated intravascular coagulation, nosocomial infections and neurological symptoms had a statistically significant worse survival [23]. In pediatric patients with secondary HLH/MAS, thrombocytopenia was a predictor of mortality [15].

HLH is characterized by a hyperinflammatory phenotype [24, 25]. As clinical criteria for HLH include non-specific findings that overlap with other diseases, HLH may be an obsolete term that is better replace by hyperinflammatory syndrome and/or hypercytokinemia [26]. The HLH-94 treatment protocol includes 8 weeks of initial therapy that aims to achieve clinical remission, followed by continuation therapy that aims to keep patients alive and stable until an acceptable bone marrow transplantation donor becomes available [4]. In the present study, 12 (13.6%) patients received dexamethasone alone and 3 (3.4%) patients did not receive any treatment; these patients had a survival rate > 90%. These findings suggest treatment can be started before definite diagnosis in severe cases with high suspicion of HLH and progressive disease. Patients should be treated with dexamethasone. Clinical decision making relevant to etoposide administration should consider disease progression, treatment effect and diagnostic criteria, which will direct treatment duration and inform prognosis. In the present study, pediatric patients with HLH who did not achieve disease control by week 8 were considered at high risk of adverse outcomes.

HLH is a rare condition, and rigorously conducted prospective studies are lacking. There remains an unmet need to identify prognostic factors for secondary HLH, as few data are available for risk-stratification. This study identified clinical symptoms, treatment response and prognostic factors in children with non-malignancy associated secondary HLH, but it was associated with several limitations. First, it was a retrospective study; despite this, medical records showed that patients were consistently treated and managed, and the sample size was adequate. Second, the risk-stratification model should be validated by larger studies. Third, there was significant heterogeneity in this cohort, in terms of attributed causes but also disease severity, since a number of patients were not treated. However, exclusion of cases of MA-HLH ensured that treatment response to the protocols and survival outcomes were applicable to risk- stratification.

## Conclusions

Findings from this study suggest that clinicians should be aware of predictive factors at diagnosis, including hemoglobin < 60 g/L and albumin < 25 g/L, and consider treatment response at 8-weeks as a criterion to identify patients with non-malignancy associated secondary HLH who are at high-risk of disease progression and in need of second-line therapy and allogeneic HCT.

## Data Availability

Data are available from the authors upon reasonable request.

## Abbreviations

HLH: Hemophagocytic lymphohistiocytosis
ALL: Acute lymphoblastic leukemia
AML: Acute myeloblastic leukemia
IVIG: Intravenous Immunoglobulin
Allo HSCT: Allogeneic Hematopoietic stem cell transplantation
OS: Overall survival
MA-HLH: Malignancy-associated HLH
CBC: Complete blood cell
PT: Prothrombin time
AST: Aspartate aminotransferase
ALT: Alanine aminotransferase
LDH: Lactate dehydrogenase
MAS: Macrophage activation syndrome
